# Gasotransmitters: Potential Therapeutic Molecules of Fibrotic Diseases

**DOI:** 10.1155/2021/3206982

**Published:** 2021-09-20

**Authors:** Yingqing Chen, Shuo Yuan, Yuying Cao, Guangyao Kong, Feng Jiang, You Li, Qi Wang, Minli Tang, Qinggao Zhang, Qianqian Wang, Liping Liu

**Affiliations:** ^1^Chronic Disease Research Center, Medical College, Dalian University, Dalian, 116622 Liaoning, China; ^2^Engineering Technology Research Center for the Utilization of Functional Components of Organic Natural Products, Dalian University, Dalian, 116622 Liaoning, China; ^3^Key Laboratory of Natural Medicines of the Changbai Mountain, Ministry of Education, College of Pharmacy, Yanbian University, Yanji, 133002 Jilin Province, China

## Abstract

Fibrosis is defined as the pathological progress of excessive extracellular matrix (ECM), such as collagen, fibronectin, and elastin deposition, as the regenerative capacity of cells cannot satisfy the dynamic repair of chronic damage. The well-known features of tissue fibrosis are characterized as the presence of excessive activated and proliferated fibroblasts and the differentiation of fibroblasts into myofibroblasts, and epithelial cells undergo the epithelial-mesenchymal transition (EMT) to expand the number of fibroblasts and myofibroblasts thereby driving fibrogenesis. In terms of mechanism, during the process of fibrosis, the activations of the TGF-*β* signaling pathway, oxidative stress, cellular senescence, and inflammatory response play crucial roles in the activation and proliferation of fibroblasts to generate ECM. The deaths due to severe fibrosis account for almost half of the total deaths from various diseases, and few treatment strategies are available for the prevention of fibrosis as yet. Recently, numerous studies demonstrated that three well-defined bioactive gasotransmitters, including nitric oxide (NO), carbon monoxide (CO), and hydrogen sulfide (H_2_S), generally exhibited anti-inflammatory, antioxidative, antiapoptotic, and antiproliferative properties. Besides these effects, a number of studies have reported that low-dose exogenous and endogenous gasotransmitters can delay and interfere with the occurrence and development of fibrotic diseases, including myocardial fibrosis, idiopathic pulmonary fibrosis, liver fibrosis, renal fibrosis, diabetic diaphragm fibrosis, and peritoneal fibrosis. Furthermore, in animal and clinical experiments, the inhalation of low-dose exogenous gas and intraperitoneal injection of gaseous donors, such as SNAP, CINOD, CORM, SAC, and NaHS, showed a significant therapeutic effect on the inhibition of fibrosis through modulating the TGF-*β* signaling pathway, attenuating oxidative stress and inflammatory response, and delaying the cellular senescence, while promoting the process of autophagy. In this review, we first demonstrate and summarize the therapeutic effects of gasotransmitters on diverse fibrotic diseases and highlight their molecular mechanisms in the process and development of fibrosis.

## 1. Introduction

Fibrosis is a well-known pathological process in which several extracellular matrixes (ECMs), such as collagen, fibronectin, and elastin, accumulate abnormally in chronic inflamed and damaged tissues. Excessive fibrosis, in chronic inflammation, can cause permanent scars, multiple organ sclerosis, and dysfunction [1]. Tissue fibrosis will be the main cause of disability and death in many diseases and which affects various organs such as the cardiovascular, lung, liver, and kidney [2].

Although the mechanisms of various fibrotic diseases are different, the common feature of tissues affected by fibrosis is the presence of excessive activated fibroblasts and transformed myofibroblasts [3]. These cells have unique biological functions, including the secretion of fibrous type I and type III collagen and the expression of *α*-smooth muscle actin (*α*-SMA) [3]. In the occurrence and development of fibrosis, the activation of fibroblast and transformation into myofibroblasts are two very important factors. Once fibroblasts are activated by tissue damage and chronic inflammation, it will secrete a variety of ECM and promote the conversion of fibroblasts to myofibroblasts. The critical molecules and signaling pathways involved in fibrosis mainly consist of transforming growth factor *β* (TGF-*β*), connective tissue growth factor (CTGF/CCN2), platelet-derived growth factor (PDGF), endothelin 1 (ET-1), and the Wnt, Hedgehog, and Notch signaling pathways [4–10]. Therefore, the strategies for the treatment of fibrosis, to date, mainly include the inhibition of fibroblast activity and the myofibroblast transformation, interfering TGF-*β* expression and its signaling pathways, decreasing the homing of fibroblasts into the tissues, and the inhibition of other profibrosis signaling pathways [11].

Gasotransmitters are small molecules with a short half-life and play crucial roles in cellular homeostasis [12]. Nitrogen monoxide (NO), carbon monoxide (CO), and hydrogen sulfide (H_2_S) are three best-known gasotransmitters, and all these three gases have been demonstrated to possess antioxidative, anti-inflammatory, antiapoptotic, and antiproliferative properties [12]. Besides that, these gasotransmitters are gradually being known by many researchers for exploring their molecular mechanisms in fibrotic diseases. In this review, we first try to discuss and summarize the therapeutic mechanisms of gasotransmitters in diverse fibrotic diseases.

## 2. Molecular and Cellular Mechanisms of Fibrosis

### 2.1. TGF-*β* and Fibrosis

TGF-*β* has a dimeric structure and is expressed in all cells. It is important for regulating cell growth, migration, immunosuppression, and endothelial- mesenchymal transition (EMT) [13]. TGF-*β*1 is the most abundant subtype of the TGF-*β* family. It plays a critical role in promoting fibrotic cell proliferation, collagen secretion, protease inhibitor production, and extracellular matrix (ECM) deposition in tissues [13]. After its receptors are bound, the signal can be further transmitted to the downstream Smads protein family [13, 14]. First, TGF-*β*1 recognizes and combines with TGF-*β*RII dimer and then forms a heterotetraploid with the TGF-*β*RI dimer successively, so that the glycine-serine- (GS-) rich region of TGF-*β*RI starts to phosphorylate and activate TGF-*β*RI kinase. Then, the activated Smad2/3 components bind with Smad4 to form oligomeric complex and which translocate into the nucleus and participate in the transcription of target genes associated with the process of cell apoptosis, proliferation, and differentiation [15]. Besides that, Smad2/3 phosphorylation can increase the expression of fibrosis relative genes and enhances the activity of fibroblasts to facilitate the progression of fibrosis [15]. However, Smad7, as an inhibitory Smad (I-Smad), may block the TGF-*β*1/Smads signaling pathway by interfering with the activated TGF-*β*RI. This also means that the phosphorylation of Smad2/3 is hindered from the root cause, thereby delaying or improving the process of fibrosis [16, 17]. Moreover, the screening of targets related to the TGF-*β* signaling pathway has become a novel method to provide theoretical support for the development of therapeutic drugs of fibrotic diseases [13–15, 18].

More recently, several studies have found that galectin-3 is a key signal molecule in the TGF-*β* pathway and which mediates fibroblast activation to provoke TGF-*β* expression and to activate its downstream profibrosis-related pathways [18]. Galectin-3 is expressed and secreted by inflammatory cells and that can bind to glycoproteins and glycolipids on cell surface to participate in a variety of physiological and pathological processes [19]. Galectin-3 has the function of accelerating the proliferation and differentiation of cardiac fibroblasts and which plays an important role in the pathophysiological process such as cardiac fibrosis. It is also an emerging marker used to diagnose and predict congenital hepatic fibrosis (CHF) in recent years [19].

### 2.2. Oxidative Stress and Fibrosis

Oxidative stress is referred as the excessive free radicals that are produced under the stimulation of harmful factors in vitro and in vivo. The excessive accumulation of oxygen free radicals and related metabolites can cause the cell to produce a variety of toxic effects [20]. Glutathione (GSH) and superoxide dismutase (SOD) are the main players in antioxidant defense system, which can eliminate harmful peroxidation metabolites, block lipid peroxidation, and protect the integrity of cellular membrane [21].

NADPH oxidase (NOX) is a transmembrane complex composing multiple protein subunits which are the main regulators of reactive oxygen species (ROS) production in cells. There are 7 subtypes of NOX, namely, NOX1, NOX2, NOX3, NOX4, NOX5, DUOX1, and DUOX2 [22]. Hepatic stellate cells (HSCs) and Kupffer cells are the key effectors for liver NOX expression. Kupffer cells mainly participate in the generation of ROS in the early stage of liver fibrosis by expressing NOX2 [23]. The increase of NOX2 can induce HSC activation by releasing ROS to mediate liver damage and fibrosis through activating the platelet-derived growth factor (PDGF) signal pathway [24].

Oxidative stress and the antioxidant system appear to be crucial modulators in regulating the TGF-*β*_1_ signaling, metabolic homeostasis, and chronic inflammation, all of which are associated with the development and persistence of fibrosis [25]. The TGF-*β*_1_/p38 MAPK signaling pathway is activated in response to various inflammations, oxidative stress, and other stimuli. Inhibiting the activity of this pathway can slow down the progression of fibrosis [26]. TGF-*β*_1_ is activated after receiving various stimuli, including oxidative stress, which further affects the activation of the downstream factor apoptosis signal-regulating kinase 1 (ASK-1). Overexpression of ASK-1 can promote MKK3/6 phosphorylation and which subsequently activate p38 MAPK and phosphorylation of cyclic AMP-dependent transcription factor 2 (ATF2), thereby producing a series of biological reactions [27]. ATF2 is a member of the leucine zipper family of DNA-binding proteins. It is mainly involved in the regulation of cellular stress response. Phosphorylated ATF2 plays a key role in inflammation, apoptosis, and fibrosis [28–31]. JNK-dependent phosphorylation of ATF2/c-Jun transcription factors can result in TGF-*β* transcription to promote oral submucous fibrosis [30]. In addition, anti-ATF2 antibody, as a novel autoantibody, can serve as a serological marker for inflammation and lung involvement in systemic sclerosis [31].

ROS production can activate the p38 MAPK pathway and that in turn aggravates the oxidative stress state, resulting in oxidative damage and tissue fibrosis [32]. Moreover, oxidative stress interacts with other pathophysiological mechanisms to promote the occurrence and development of fibrosis [33]. Therefore, the role of oxidative stress in fibrotic diseases has been attracted more attention by researchers. Only clarifying the complex mechanism can we discover the effective antioxidative stress drugs to treat fibrosis.

### 2.3. Cellular Senescence and Fibrosis

Numerous studies have strongly suggested that the progression of fibrotic diseases is highly correlated with age. The accumulation of senescent cells caused by aging is proved as a key factor to the development of fibrosis [34]. With the aging of the body, the increase of damaged cells and the decline in immune monitoring capabilities can reduce the clearance rate of senescent cells and which subsequently result in the secretion of various senescence-associated secretory phenotypes (SASPs), such as proinflammatory cytokines, chemokines, and metalloproteinases (MMPs), to accelerate the development of fibrosis [35, 36].

The activation of p53, after the senescence occurred in type II alveolar epithelial cells, can participate in the development of pulmonary fibrosis by upregulating miR-34 and downregulating key target genes of the cell cycle [37]. Moreover, p21 can maintain the survival of senescent HSCs by inhibiting caspase and c-Jun N-terminal kinase (JNK) signals [38]. Lehmann et al. proved that the type II alveolar epithelial cells from mice with pulmonary fibrosis could secrete higher levels of SASP, especially insulin-like growth factor-binding protein- (IGFBP-) 3, 4, 7 and matrix metalloproteinase- (MMP-) 3, 12, 14 [39]. Therefore, prevention of cellular senescence is of great significance for attenuating the pathogenesis of fibrosis and which can provide novel antifibrotic treatment strategies.

Recent studies have found that the TGF-*β* pathway is also involved in the aging process. Tasanarong et al. suggested that TGF-*β* could trigger stress-induced senescence via the p16^INK4a^ and Smad3 pathways, and the loss of Smad3 will reduce the formation of senescent cells [40, 41]. Furthermore, in the process of cellular senescence, senescent cells are resistant to cell apoptosis, and whose accumulation over time can secrete a variety of SASP and which subsequently stimulate fibroblasts into damaged tissues, as well as accelerate the activation of fibroblasts [40]. Fang et al. demonstrated that advanced glycation end-products (AGE) are an important factor for cardiac aging and fibrosis, and AGE-induced cardiac aging might be the crucial factor for TGF-*β*-mediated fibrosis [42].

Conversely, studies have shown that after knocking out p53, HSCs could continuously promote the expression of ECM and *α*-SMA to accelerate the process of liver fibrosis [43]. This indicated that the senescent HSCs could interfere with the development of liver fibrosis [43]. In addition, insulin-like growth factors (IGF-I) can induce HSC senescence. IGF-I can increase the expression of senescence-associated proteins, such as p21 and p53, to reduce ECM deposition and prevent liver fibrosis [43, 44]. Kong et al. found that IL-22 can induce HSC senescence through the p53 pathway and which can eventually reverse liver fibrosis in mice [45]. These studies indicate that senescent HSCs can inhibit the development of liver fibrosis through activating the p53 signaling pathway and which are expected to realize the reversal of liver fibrosis [43–45].

### 2.4. Inflammation and Fibrosis

In most chronic inflammatory diseases, fibrosis has increasingly become the main cause of morbidity and mortality [46]. Many immune-related elements are involved in the occurrence and development of fibrotic diseases [47, 48].

Nuclear factor-*κ*B (NF-*κ*B) is a transcriptional regulatory factor that has been studied extensively in recent years [49]. Translocation of activated NF-*κ*B into the nucleus can promote the expression of related genes, such as TNF-*α*, IL-6, IL1*β*, and NLRP3 [49]. These substances are reported to play an important role in the occurrence and development of fibrosis [50–55]. Research showed that Krüppel-like factor 4 (KLF4), a zinc finger transcription factor, can ameliorate chronic kidney disease through mitigating TNF-mediated tissue injury and fibrosis [50]. Moreover, TNF-*α* can stimulate IL-33 secretion via interaction with TNFR2 and which promotes myofibroblast development to accelerate the process of myocardial fibrosis [51]. NOD-like receptor protein 3 (NLRP3) inflammasome inhibitor, MCC950, is first demonstrated to ameliorate nonalcoholic fatty liver disease (NAFLD) and fibrosis in obese diabetic mice, and the targeting of NLRP3 is a logical direction in pharmacotherapy for liver fibrosis [54].

**Figure 1:**
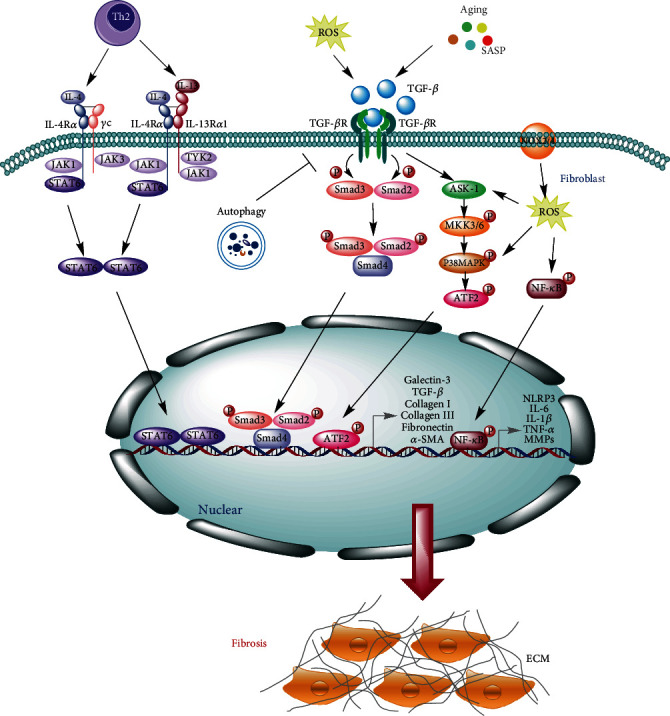
Molecular and cellular mechanisms of fibrosis. TGF-*β* recognizes and combines with TGF-*β*R II and TGF-*β*R I successively, and then, the glycine-serine- (GS-) rich region of TGF-*β*R I phosphorylates Smad2/3 to facilitate the formation of oligomeric complex with Smad4 and which translocates into the nucleus and participates in the transcription of fibrotic genes, such as galectin 3, collagen I, collagen III, *α*-SMA, and TGF-*β*. Besides that, the TGF-*β* signaling pathway can be activated by oxidative stress and cellular senescence, which further affects the activation of the downstream factors ASK-1. ASK-1 can promote MKK3/6 phosphorylation and which subsequently activates p38 MAPK and phosphorylates ATF2, thereby promoting the transcription of fibrosis-associated genes. NOX2/4 can stimulate ROS production and provoke the NF-*κ*B signaling pathway to upregulate inflammatory-associated genes, such as NLRP3, IL-6, IL-1*β*, TNF-*α*, and MMPs, to promote the development of fibrosis. IL-4 and IL-13 secreted by Th2 cells can activate STAT6 and which can promote the expression of fibrotic genes and inflammatory cytokines to accelerate the process of fibrosis. Autophagy exerts a protective role in fibrotic diseases by downregulating the TGF-*β*/Smad4 pathway and NLRP3 inflammasome.

Signal transducers and activators of transcription 6 (STAT6) is mainly activated by IL-4 and IL-13 and plays an important role in immune regulation, involving the development of fibrosis [56, 57]. STAT6 is mainly activated by cytokines, including IL-4 and IL-13, which are secreted by Th2 cells (Figure 1). IL-13 is an important fibrotic factor. The profibrosis effect of IL-13 may be closely related to the upregulation of many fibrosis-related proteins in HSC and HSC activation. IL-13 can regulate liver fibrosis by activating macrophages. Both the upregulation of miR-142-5p and the downregulation of miR-130a-3p in macrophages can promote fibrosis [56]. The upregulated miR-142-5p promotes the phosphorylation of STAT6 by targeting SOCS1, and the downregulated miR-130a-3p reduces its inhibition of peroxisome proliferator-activated receptor *γ* (PPAR *γ*) to promote fibrosis [56]. In the bleomycin-induced mouse pulmonary fibrosis model, IL-4 and IL-13 levels were significantly increased, but after blocking the IL-4/IL-13 signaling pathway, the activation of STAT6 was reduced and which could significantly ameliorate pulmonary fibrosis [57].

Autophagy is a highly conservative cell degradation and recycling process that can regulate cell death and proliferation. Previous studies have shown that autophagy has an inhibitory effect in fibrosis (Figure 1) [58]. Conditionally knocking out the autophagy-related protein 7 (Atg7) gene in the distal renal tubular epithelial cells from unilateral ureteral obstruction (UUO) mouse model can promote the activation of the TGF-*β*/Smad4 pathway and the NLRP3 inflammasome, as well as aggravate renal interstitial fibrosis [58]. This suggests that autophagy can play a protective role in renal interstitial fibrosis by regulating the TGF-*β*/Smad4 pathway and NLRP3 inflammasome.

Hypoxia can induce an increase of NLRP3 inflammasome. NLRP3 gene knockout renal tubular cells can reduce the production of ROS under hypoxic conditions. ROS scavengers can downregulate the expression of NLRP3 and reduce renal fibrosis [59]. Although the NLRP3 inflammasome has been reported to associate with the process of fibrosis, the exact mechanisms have not been fully clarified, and further studies are required to identify and provide novel possibilities for the treatment of fibrosis.

The abnormal expression of various inflammatory components in fibrotic diseases suggests that the inflammation is an important link in the occurrence and development of fibrosis in various organs. Therefore, the increasing understanding of inflammation-related signaling pathways will provide new therapeutic ideas and support more direct and effective drug targets for the treatment of fibrotic diseases.

## 3. Protective Effects of Gasotransmitters in Fibrotic Diseases

### 3.1. The Interference Role of NO in Fibrotic Diseases

**Figure 2:**
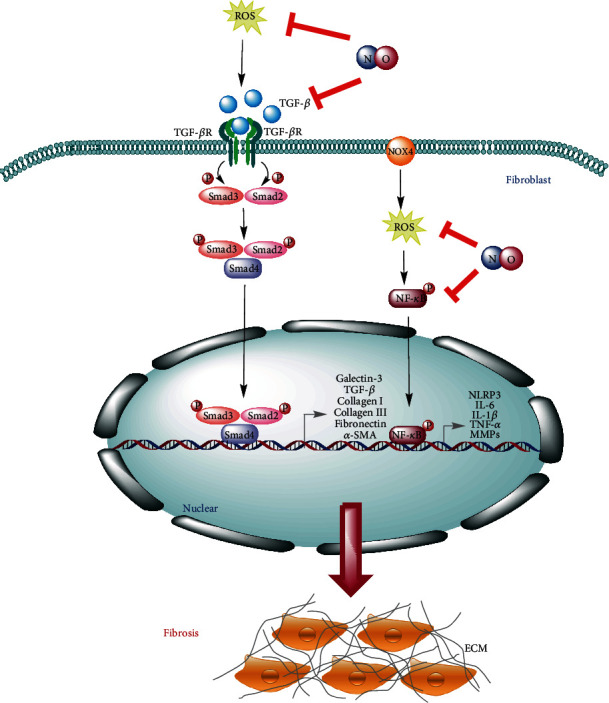
The interference mechanisms of NO in fibrotic diseases. NO can reduce the amount of ROS by peroxynitrite formation and which subsequently attenuates the activation of the NF-*κ*B signaling pathway to inhibit the expression of fibrosis and inflammatory-related genes. Moreover, NO can also downregulate the expression of TGF-*β* to attenuate its downstream signaling pathway.

Nitric oxide has been studied in many medical areas and which is defined as an important player in most physiological systems, such as nervous, cardiovascular, and conventional outflow physiology [60–63]. Endogenous NO is mainly produced intracellularly by the enzymatic action of NO synthase (NOS) from amino acid L-arginine. The three different types of NOS mainly include neuronal NOS (nNOS or NOS_1_), inducible NOS (iNOS or NOS_2_), and endothelial NOS (eNOS or NOS_3_) [64]. NO has a short half-life and is rapidly transformed into stable final products in the body, such as nitrate (NO_3_) and nitrite (NO_2_) [64]. NO has a variety of biological functions, including relaxing smooth muscle, lowering blood pressure, inhibiting the proliferation of vascular smooth muscle cells, preventing the aggregation of platelets, and enhancing nonspecific immune defense [65]. Recently, increasing studies demonstrated that the endogenous and exogenous of NO showed protective effects on diverse fibrotic diseases via multiple antifibrotic mechanisms (Figure 2) [66–75].

#### 3.1.1. NO and Liver Fibrosis

Liver fibrosis is a dynamic response process to various stimuli, such as alcoholism, viral infection, and toxins, which can lead to the destruction of liver parenchymal structure and excessive deposition of the extracellular matrix, thereby promoting the formation of liver fibrosis [76]. Long-term lack of treatment can further cause liver cirrhosis and hepatocellular carcinoma. Liver fibrosis is characterized by sustained activation of hepatic stellate cells (HSCs) and the excessive accumulation of ECM [76].

ROS is widely known to play a critical role in the development of liver fibrosis, and NO can react with ROS to produce peroxynitrite, which is normally recognized as a very reactive, toxic, and strongly oxidizing compound [66]. However, the relative amounts of peroxynitrite can act as a scavenger of ROS and which depend on the exact conditions of the local microenvironment. Svegliati-Baroni et al. [66] have demonstrated that the supplementation of exogenous NO donor, S-nitroso-N-acetylpenicillamine (SNAP), could prevent liver cirrhosis by scavenging the production of ROS, thus inhibiting HSCs activation and proliferation.

NO derived from eNOS in liver sinusoidal endothelial cells (LSECs) is demonstrated to possess a protective effect on the development of fibrotic disease. In pathological conditions, LSECs become dysfunctional, and the level of NO produced by eNOS showed a significant decrease followed by the activation of quiescent HSCs, and leading to the deposition of the ECM, fibrogenesis, and further cirrhosis [67, 77]. Simvastatin, one kind of lipid-lowering medication, was reported to provoke a conversion of activated HSCs into quiescent cells via enhancement of eNOS mediated by transcription factor Krüppel-like factor 2 (KLF2) [78]. Langer DA suggested that NO could limit the activation of HSCs by promoting the apoptosis of HSCs and which was conferred by mitochondrial membrane depolarization but not the caspase-dependent pathway [79].

As opposed to the protective effect of eNOS on fibrogenesis, iNOS exerts an inducing effect in the occurrence and development of liver fibrosis [68, 69]. The deletion or mutation of the iNOS can reduce the development of liver fibrosis [68]. Anavi et al. showed that as iNOS gene knockout mice were fed with a high-cholesterol diet for 6 weeks, the liver fibrosis was significantly attenuated compared with that in wild-type mice, and the expressions of inflammatory cytokines and fibrogenic genes were all remarkably decreased. However, the paradoxical mechanisms of NOS in liver fibrosis remain unclear [68].

#### 3.1.2. NO and Renal Fibrosis

Renal fibrosis is the buildup of scar within renal parenchyma and which commonly occurs in all chronic and progressive nephropathies, including glomerular hyperfiltration, hyperperfusion, high pressure, and ischemia-reperfusion injury [80]. The basic pathological features of renal fibrosis are composed of the injury and death of renal parenchymal cells, the infiltration of interstitial inflammatory cells, the proliferation of fibroblasts, the transformation of myofibroblasts, the excessive deposition of the ECM, and the formation of interstitial fibrosis [80].

Glomerulonephritis is a type of kidney disease in which there is inflammation of the glomeruli and progresses through the accumulation of ECM and results in loss of the glomerular architecture and scarring [81]. The pathogenesis of glomerulonephritis is incompletely understood; however, increasing studies indicated that the immunological injuries of resident cells in glomeruli, such as mesangial cells and podocytes, were associated with the focal glomerulosclerosis [82]. In the process of cultured rat glomerular mesangial cells (MCs), the administration of NO donors, such as spermine NONOate, NOC-18, and SNAP, can suppress the expression of fibrogenic genes at the transcriptional level [70]. This study revealed a complex role of NO in regulating gene expression in mesangial cells and suggests an antifibrotic potential of NO. Additionally, Peters et al. revealed that the supplementation of L-arginine can reduce the fibrotic disease in antithymocyte serum- (ATS-) glomerulonephritis animal model and which is mediated by multiple pathways, including the suppression of TGF-*β* expression, while further studies are required to reveal the therapeutic potential of L-arginine supplementation in humans [72, 73].

MMP-9 is an essential matrix metalloproteinase involved in the process of renal fibrosis, which can be regulated in different levels, and finally, the endogenous MMP-9 inhibitor TIMP-1 inhibits the enzyme activity. In the kidney, glomerular mesangial cells are the main source for the synthesis of MMP-9 and its endogenous inhibitor TIMP-1. In renal MCs, NO can modulate the expression of several ECM-degrading proteases and intrinsic inhibitors, including MMP-9, MMP-13, plasminogen activator inhibitor-1 (PAI-1), and TIMP-1 [83, 84]. In addition, NO donor SNAP can amplify the expression of TIMP-1 in a TGF-*β*-dependent manner and thereby may play a critical role in the regulation of the proteinase-antiproteinase homeostasis in renal MCs [71].

#### 3.1.3. NO and Idiopathic Pulmonary Fibrosis

Idiopathic pulmonary fibrosis (IPF) is a chronic and progressive interstitial lung disease, which can be triggered by various harmful factors like toxic chemicals, radiation, inorganic particles, and microbial infections. Its basic pathological process is the activation of lung fibroblasts and the transformation into myofibroblasts and which can cause the excessive deposition of ECM and ultimately destruct the lung parenchymal structure. The typical pathological features of idiopathic pulmonary fibrosis are the proliferation and accumulation of lung parenchymal fibers and their structural destruction [85].

Patients with pulmonary fibrosis showed a significant enhancement of NOS expression and plasma nitrite and nitrate (NOx) levels, indicating that NO might play an important role in the process of lung fibrosis [86–88]. The use of bleomycin can induce pulmonary fibrosis in mice. In the lack of all three NOS isoforms, including iNOS, eNOS, and nNOS, bleomycin-treated mice showed a deterioration of pulmonary fibrosis, suggesting the entire endogenous NO and NOS systems exert an important protective role in the pathogenesis of pulmonary fibrosis [74].

Cyclooxygenase-inhibiting NO donors (CINODs) are designed to inhibit COX1 and COX2 while releasing NO, which exhibits anti-inflammatory, pain-relieving, and antioxidant effects [75]. In bleomycin-induced lung fibrosis model, one prototype CINOD compound, (S)-(5S)-5,6-bis(nitrooxy)hexyl)2-(6-methoxynaphthalen-2-yl) propanoate (NCX466), has shown a significant efficacy in reducing lung inflammation, the TGF-*β* signaling pathway, and the collagen accumulation, suggesting that COX inhibition along with NO donation may exhibit therapeutic potential in pulmonary fibrosis [75].

#### 3.1.4. NO and Peyronie's Disease

In normal condition, myofibroblasts share both phenotypic characteristics of fibroblasts and smooth muscle cells. It plays a key role in collagen deposition and wound healing and disappears through apoptosis when the wound is healed. However, its abnormal persistence can be observed in the fibrotic plaque of the tunica albuginea (TA) of the penis in men with Peyronie's disease (PD) [89, 90]. Vernet demonstrated that the enhancement of iNOS via the administration of cytokine cocktail plus NO donor, SNAP, can inhibit the process of fibrosis by the reduction of myofibroblast abundance and lead to a reduction in collagen 1 synthesis and the inhibition of ROS production [91].

### 3.2. The Interference Role of CO in Fibrotic Diseases

Carbon monoxide (CO) is generated by the action of heme oxygenase (HO), which is involved in the degradation of heme [92]. Although the toxic effects of CO are well documented, it was only discovered in the last decade that low concentration of CO can exert numerous biological effects, such as anti-inflammation, antiapoptosis, and antioxidation [93] As the sudden surge in research of CO and its beneficial biological effects, several novel compounds termed carbon monoxide-releasing molecules (CORMs) have been developed, and their biochemical properties have been characterized [94, 95]. The two most recently developed CORM are tricarbonylchoro(glycinato)ruthenium (II) (CORM-3) and sodium boranocarbonate (CORM-A1), both of which are fully water-soluble and thus easy to handle. Upon incubation in a physiological medium, both CORM-3 and CORM-A1 can liberate CO gas [95].

Aki et al. demonstrated that the application of 1 mM CORM3 to mouse embryonic fibroblasts (MEFs) resulted in the reduction of collagen I and III within 24 h, which confirmed an antifibrotic effect of CO [96] CORM3 also caused a rapid dissociation of cell-associated plasma fibronectin (FN) from MEFs within 1 h, and which is associated with the formation of a reduction-resistant oligomer of plasma FN, suggesting FN is a CORM-3-interactive plasma protein, and the CORM-3-FN interaction is involved in the death of fibroblasts [96].

#### 3.2.1. CO and Idiopathic Pulmonary Fibrosis

Zhou et al. reported that in bleomycin-induced IPF mouse model, inhaling low-dose exogenous 250 ppm CO gas into mice showed a significant therapeutic effect on the development of lung fibrosis [97]. This study revealed that CO could inhibit the formation of pulmonary fibrosis by inhibiting the synthesis and the deposition of extracellular matrix, as well as the proliferation of fibroblasts through increasing p21Cip1 expression while decreasing cyclin A and D levels. Furthermore, CO-exposed cells significantly downregulated fibronectin (FN) and collagen-1 via the regulation of transcriptional regulator, an inhibitor of DNA binding 1 (Id1) [97]. Besides that, one recent study also demonstrated that a nanotechnology-based CO donor, CO-bound hemoglobin vesicles (CO-HbV), showed a therapeutic effect on IPF and which was attributed to a decrease in ROS production by the NOX4 signaling pathway, as well as the production of inflammatory cytokines and TGF-*β* in the lung [98]. Rosas et al. have demonstrated that inhaled low-dose CO gas was well tolerated and can be safely administered to patients with IPF in phase II clinical trials [99]. Overall, exogenous and endogenous CO exert an antifibrotic effect in the lung, and this effect can ameliorate bleomycin-mediated IPF.

#### 3.2.2. CO and Renal Fibrosis

Wang and his colleagues reported that the exogenous administration of CO can ameliorate UUO-induced renal fibrosis and protect against kidney injury [100]. As mice were exposed to CO, the deposition of ECM and the expression of *α*-SMA, type I collagen, and FN in the kidneys were significantly decreased. In addition, the beneficial effect of CO is mainly associated with the MKK3 pathway. These findings suggest that low-dose CO exerts protective effects on inhibition of renal fibrosis in obstructive nephropathy [99].

#### 3.2.3. CO and Myocardial Fibrosis

Myocardial fibrosis refers to the pathological process in which various harmful stimuli, such as myocardial injury, mechanical stretching, and myocardial inflammation, trigger the proliferation of fibroblasts in heart tissue. This process leads to excessive ECM deposition and disorganization of cardiac structure and function, such as cardiac hypertrophy, heart failure, and arrhythmia [101].

**Figure 3:**
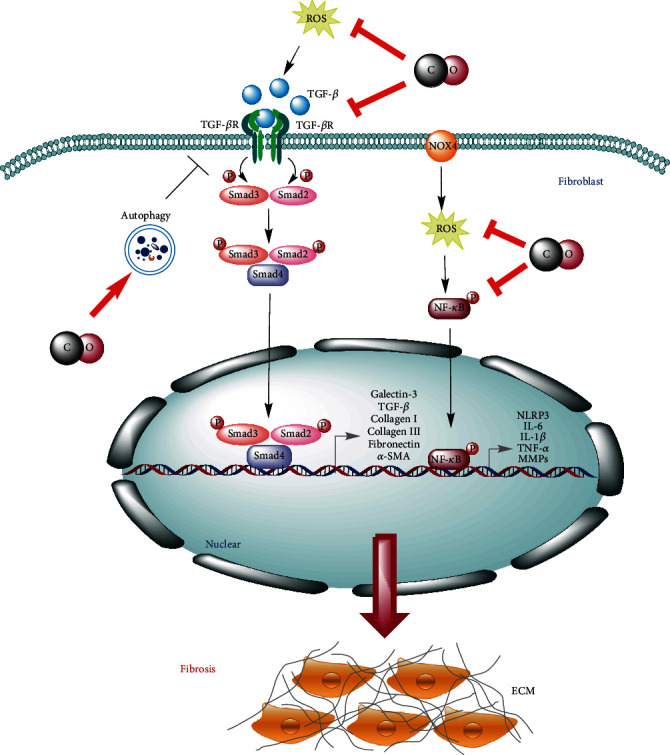
The interference mechanisms of CO in fibrotic diseases. Low-dose exogenous and endogenous CO can interfere with the TGF-*β* and NF-*κ*B signaling pathway via reducing the expression of fibrosis and inflammatory-related genes. In addition, CO can also inhibit the TGF-*β* signaling by the stimulation of autophagy.

Human immunodeficiency virus (HIV) protease inhibitor-induced cardiac dysfunction is characterized as a pathologic fibrosis related to the activation of TGF-*β*_1_ [102, 103]. Laurence et al. have demonstrated that inhalation of low-dose CO (250 ppm) can suppress ritonavir-induced cardiac fibrosis and which is modulated by the canonical (Smad2) and noncanonical TGF-*β*_1_ signaling pathways. In addition, CO treatment can also suppress the M1 proinflammatory subset of macrophages, while increase M2c subset of macrophages in the hearts of ritonavir-treated mice and which is also associated with CO-induced autophagy [104]. Taken together, the antifibrotic effects of CO are linked to the inhibition of the TGF-*β* signaling and the stimulation of autophagy as shown in Figure 3.

### 3.3. The Interference Role of H_2_S in Fibrotic Diseases

**Figure 4:**
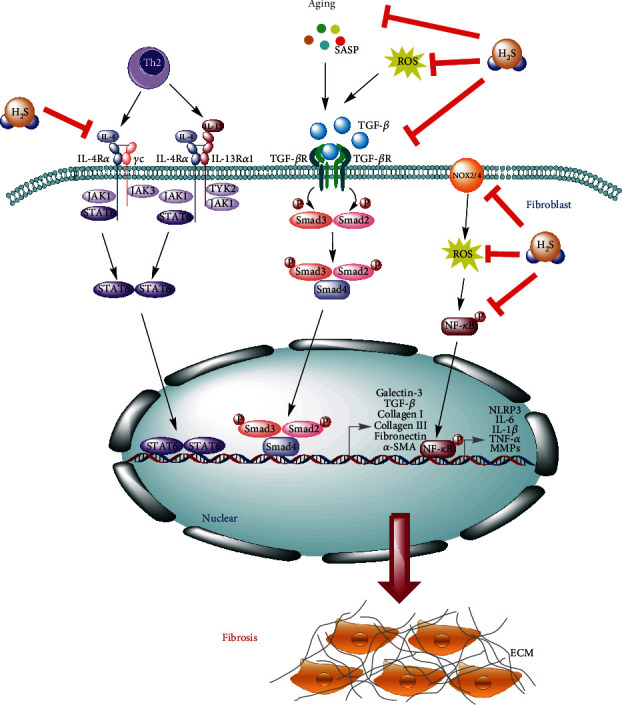
The interference mechanisms of H_2_S in fibrotic diseases. H_2_S can effectively slow down the process of fibrosis through inhibiting the NF-*κ*B, TGF-*β*, and IL-4/STAT6 signaling pathways, as well as attenuating the production of ROS via downregulation of NOX2/4. Moreover, H_2_S can also inhibit the formation of NLRP3 inflammasome and the process of aging to control the occurrence and development of fibrosis.

Hydrogen sulfide is a widely known gas with a malodorous smell and which is the most recently recognized member of gaseous signaling molecules, as well as exhibits remarkable therapeutic characteristics in several pathologies [105]. H_2_S is produced endogenously in the cytoplasm and mitochondria of mammalian cells by utilizing L-cysteine and D-cysteine as substrates catalyzed cystathionine-*β*-synthase (CBS), cystathionine-*γ*-lyase (CSE), 3-mercaptopyruvate sulfurtransferase (3-MST), and D-amino acid oxidase (DAO) [106–108]. Besides its endogenous production, H_2_S can also be produced via its exogenous sources such as sodium hydrosulfide (NaHS), sodium sulfide (Na_2_S), S-allyl-cysteine (SAC), GYY4137, AP39, and AP123, SG1002, S-propargyl-cysteine, sodium thiosulfate, sulfurous mineral water, garlic-derived polysulfide, diallyl disulfide, and diallyl sulfide [109]. Numerous studies have shown that low-dose exogenous and endogenous H_2_S have therapeutic and protective effects on common organ fibroproliferative diseases and syndromes and which are mainly due to its anti-inflammatory, antioxidant, and antifibrotic properties (Figure 4) [110]. Furthermore, the deficiency in endogenous CBS/H_2_S or CSE/H_2_S system is involved in the development of fibrosis [111], while the supplementation of exogenous H_2_S can significantly inhibit the progression of fibrosis [112–115].

#### 3.3.1. H_2_S and Renal Fibrosis

In normal condition, the expression of CBS is predominantly located in proximal renal tubules, while a small amount of CSE is expressed in the renal glomeruli, interstitium, and interlobular arterioles. Moreover, MST is mainly expressed in the proximal tubular epithelium in the kidney [116, 117].

In unilateral ureteral obstruction- (UUO-) induced renal injury and fibrosis mouse model, treatment with H_2_S donor NaHS can significantly reduce kidney damage and fibrosis through the inhibition of M1 and M2 macrophages' infiltration and downregulation of fibrogenic genes [118]. These beneficial effects of NaHS might be contributed to the inactivation of NLRP3, as well as to its downstream signaling pathways and the phosphorylation of the NF-*κ*B and IL-4/STAT6 signaling pathways [118]. Additionally, in the recovery of the kidney following ischemia/reperfusion (I/R) injury, the levels of CSE, CBS, and H_2_S were significantly decreased and which did not recover in eight days as fibrotic lesions were observed. However, the administration of NaHS can accelerate tubular cell proliferation and delay the progression of renal fibrosis by attenuating oxidative stress and inflammation in a mouse model with ureteral obstruction [119].

Angiotensin II (Ang II) and TGF-*β* can induce renal tubular epithelial-to-mesenchymal (EMT), and the abnormal activation of EMT can lead to the tubular interstitial fibrosis. In the presence of H_2_S donor NaHS, the TGF-*β* signaling pathway and the EMT-promoting effect of Ang II were all decreased and which were contributed to the reduction of TGF-*β* activity [120]. NaHS can cleave the disulfide bond in the dimeric active TGF-*β*_1_ and subsequently promote to form inactive TGF-*β* monomer form [120]. This study provides a novel antifibrotic mechanism of H_2_S and suggests that H_2_S can be used to treat renal sclerotic diseases (Figure 4) [120]. Besides that, NaHS can also affect TGF-*β*_1_-induced EMT in renal tubular epithelial (HK-2) cells and which might be associated with suppression of both the ERK- and *β*-catenin-dependent signaling pathways to ameliorate renal fibrosis [121].

#### 3.3.2. H_2_S and Myocardial Fibrosis

In mammals, CSE is abundant in the heart, vascular smooth muscle, and vascular endothelial cells. It is the most relevant enzyme in the cardiovascular system to produce H_2_S. Increasing evidence demonstrates that endogenous H_2_S can participate in attenuating the development of myocardial fibrosis [122]. The decline of H_2_S content in heart tissues was significantly correlated with the severity of myocardial fibrosis [122–124]. Ma et al. have demonstrated that chronic aerobic exercise training can upregulate CSE and 3-MST expression. Furthermore, as aged rats were given moderate-intensity exercise or treated with NaHS (intraperitoneal injection of 0.1 mL/kg per day of 0.28 mol/L NaHS), the myocardial hydroxyproline level and fibrotic area in the heart could be declined dramatically, suggesting that exercise could restore bioavailability of H_2_S in the heart of aged rats and which partly explained the benefits of exercise against myocardial fibrosis of aged population [125].

Nicotinamide adenine dinucleotide phosphate II (NADPH) oxidase 2 (NOX2) and NADPH oxidase 4 (NOX4) are the main sources of reactive oxygen species (ROS), which play a key role in the fibrotic reaction of cardiac fibroblasts and ischemic myocardium. The exogenous treatment of NaHS and sodium thiosulfate (STS) can remarkably decrease NOX2/4, phosphorylation of ERK1/2, and the generation of ROS to ameliorate oxidative stress-mediated myocardial fibrosis [115, 122]. Moreover, the slow-releasing water-soluble H_2_S donor, GYY4137, can reduce adverse remodeling and play postischemic cardioprotective effects through the enhancement of early postischemic endogenous natriuretic peptide activation [123]. Similarly, the administration of GYY4137 can also prevent myocardial infarction-induced adverse cardiac remodeling in both wild type- and CSE-deficient mice [126]. One recent study reported that after the administration of NaHS, the area of myocardial fibrosis in myocardial infarction (MI) rats is reduced, and the level of type I collagen, type III collagen, and MMP-9 is reduced, and the heart function is improved. This study demonstrated that exogenous H_2_S potentially prevents heart remodeling by inhibition of extracellular matrix accumulation and increasing blood vessel density [124].

#### 3.3.3. H_2_S and Liver Fibrosis

In the combination of carbon tetrachloride (CCl4) and olive oil-induced liver fibrosis model, the treatment of S-allyl-cysteine (SAC, 50 mg/kg/day), one endogenous donor of H_2_S, could significantly reduce the mRNA expression of inflammatory and fibrotic cytokines, as well as increased the levels of antioxidant relative genes, including superoxide dismutase, catalase, and glutathione peroxidase [127]. Moreover, the treatment of SAC can also decrease the phosphorylation of Smad3 and STAT3, as well as further inhibit their ability to bind to transcription promoters. Therefore, the exogenous donor of H_2_S, SAC, can reduce CCl4 and olive oil-induced liver fibrosis through its antioxidant and anti-inflammatory features, as well as the inhibition of the STAT3/SMAD3 signaling pathway to control fibrotic gene expression [127].

Several studies indicated that plasma levels of H_2_S exhibited a dramatic decline during the progression of hepatic fibrosis [128, 129], and the expression of H_2_S produced by CBS and CSE was significantly decreased in patients with cirrhosis-induced portal hypertension [130]. These reports suggested that the inhibition of endogenous H_2_S might be associated with the development of human and animal liver fibrosis. For that, the supplementation of NaHS could significantly elevate serum H_2_S level, prevent portal pressure, decrease hyaluronic acid and hepatic hydroxyproline levels, and reduce the number of activated HSCs by induction of G1 phase cell cycle arrest [131–133]. Conversely, one recent study found that the exogenous and endogenous H_2_S can increase the proliferation and activation of HSCs, and inhibitors of H_2_S can decrease the proliferation and fibrotic marks of HSCs, and which is mediated by the cellular bioenergetics [134]. These paradoxical reports suggested that the protective effect and the molecular mechanisms of H_2_S in hepatic fibrosis should be proved in further animal and cellular studies.

#### 3.3.4. H_2_S and Idiopathic Pulmonary Fibrosis

In one recent study, bleomycin-induced IPF mouse model revealed that the plasma concentration of H_2_S and CSE activity in lung tissues was significantly downregulated on day 7, while the injection of sodium hydrosulfide (NaHS, 1.4 and 7 *μ*mol/kg body weight) could significantly decrease the collagen deposition and the severity of pulmonary fibrosis [113]. The therapeutic effect of H_2_S in bleomycin-induced IPF could be attributed to the inhibition of NF-*κ*B p65 expression and downregulation of Th2 cells [135]. Conversely, a high level of H_2_S (50~500 ppm) can lead to bronchiolitis obliterans and pulmonary edema, ultimately leading to chronic inflammation and idiopathic pulmonary fibrosis [136]. These studies clearly suggested that H_2_S exhibits two faces of the same coin and the mechanisms by which the excessive administration of H_2_S in the promotion of IPF is still not uncovered.

#### 3.3.5. H_2_S and Diabetic Diaphragm Fibrosis

Diabetes is related to the failure of multiple organs and can lead to respiratory dysfunction by reducing the endurance of respiratory muscles and lung capacity. The diaphragm is an important skeletal muscle in the mammalian breathing process [137]. Excessive inflammation of diabetes often leads to collagen deposition and muscle fibrosis. However, one recent study demonstrated that administration of NaHS could ameliorate hyperglycemia-induced diaphragm muscle fibrosis and improved the diaphragmatic biomechanical properties in diabetes mellitus, which might be associated with the alleviation of collagen deposition through the suppression of NLRP3 inflammasome-mediated inflammatory reaction [137]. This report confirmed that the therapeutic strategies aimed at inhibiting NLRP3 inflammasome-mediated fibrosis by utilizing exogenous H_2_S might serve as efficient targeted therapy in diabetes mellitus.

#### 3.3.6. H_2_S and Peritoneal Fibrosis

Peritoneal fibrosis is one of the long-term complications of peritoneal dialysis (PD) patients [138]. Peritoneal fibrosis model was constructed in Sprague-Dawley rats through intraperitoneally injecting 4.25% glucose PD fluids and lipopolysaccharide. After daily injection of 56 *μ*g/kg NaHS, this can significantly decrease the biomarkers of inflammation, fibrosis, and angiogenesis in the peritoneum, although the exact molecular mechanisms have not been discovered [139]. The evidence suggests again that the exogenous H_2_S possess a strong antifibrotic property and which can be a potential therapy against peritoneal fibrosis during chronic PD.

## 4. Conclusion and Perspectives

**Figure 5:**
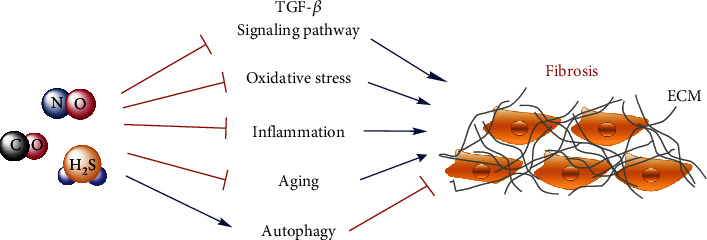
Three gasotransmitters, NO, CO, and H_2_S, can prevent the development of organ fibrosis through interfering with the TGF-*β* signaling pathway, oxidative stress, inflammation, and aging and provoking autophagy.

According to these reports, many stress responses and molecular targets are involved in the formation and development of fibrosis. Among them, the activations of the TGF-*β* signaling pathway, oxidative stress, chronic inflammation, and aging are considered as key regulatory targets (Figure 1). Three well-known gasotransmitters, NO, CO, and H_2_S, are demonstrated to regulate the development of fibrotic diseases mainly through anti-inflammation, antioxidation, antiapoptosis, and the induction of autophagy (Table 1). To date, although a great number of animal experiments have reported that these gasotransmitters exhibited various beneficial effects (Figure 5) on delaying the process of fibrotic disease, more clinical trials are necessary to be applied for proving their therapeutic effects on fibrotic diseases. In addition, to support more evidence about their preventive effects, the detailed molecular and cellular mechanisms are also needed to be further clarified. In the future study, given the synergistic effect of these gasotransmitters on fibrotic diseases, the combinatorial treatment of exogenous NO, CO, and H_2_S donors can be applied for delaying the process of fibrosis. Moreover, it is necessary to develop and utilize more effective gas donors as important components of health products for the prevention and treatment of fibrotic diseases.

## Figures and Tables

**Table 1:** Related mechanisms of gasotransmitters in fibrotic diseases.

Gasotransmitters		Diseases	Mechanisms	References
NO	SNAP	Liver fibrosis	SNAP can eliminate the generation of ROS, inhibit the activation and proliferation of HSC, and inhibit the generation of fibrosis.	[66]
		Renal fibrosis	SNAP can amplify the expression of TIMP-1 in a TGF-*β*-dependent manner and reduce fibrosis.	[71]
		Peyronie's disease	SNAP can inhibit fibrosis by inhibiting the production of ROS, decreasing the expression of collagen 1, and reducing the abundance of myofibroblasts.	[91]
	eNOS	Liver fibrosis	eNOS can reduce fibrosis by promoting HSC apoptosis and ROS-mediated mitochondrial membrane depolarization to inhibit HSC activation.	[77–79]
	L-arginine	Renal fibrosis	L-arginine can be mediated through a variety of pathways, including inhibiting the expression of TGF-*β* to reduce fibrosis.	[73]
	CINOD	Idiopathic pulmonary fibrosis	CINOD can inhibit the expression of COX1 and COX2, showing anti-inflammatory and antioxidant effects to resist fibrosis.	[75]

CO	CORM3	Activation of mouse embryonic fibroblasts	1 mM CORM3 can reduce the production of collagen I and III and interact with plasma fibronectin to prevent fibrosis.	[96]
	CO-HbV	Idiopathic pulmonary fibrosis	CO-HbV reduces the production of ROS by inhibiting the NOX4 signaling and attenuating the TGF-*β* signaling pathway.	[98]
	CO gas	Idiopathic pulmonary fibrosis	Exogenous 250 ppm CO gas inhibits the synthesis of deposition of ECM and interferes with the proliferation of fibroblasts through the regulation of Id1 expression.	[97]
		Renal fibrosis	250 ppm CO can ameliorate UUO-induced renal fibrosis and protect against kidney injury.	[100]
		Myocardial fibrosis	250 ppm CO can play an antifibrosis effect by inhibiting the TGF-*β* signaling and stimulating autophagy.	[103, 104]

H_2_S	NaHS	Idiopathic pulmonary fibrosis	NaHS can reduce the deposition of collagen and reduce pulmonary fibrosis.	[113]
		Renal fibrosis	NaHS can significantly reduce fibrosis through phosphorylation of the NF-*κ*B and IL-4/STAT6 signaling pathway and inactivation of NLRP3 and its downstream signaling pathways, inhibiting the infiltration of M1 and M2 macrophages and downregulating fibrogenic genes.	[118]
		Renal fibrosis	NaHS can accelerate the proliferation of renal tubular cells and delay renal fibrosis by reducing oxidative stress and inflammation.	[119]
		Renal fibrosis	NaHS can prevent the formation of fibrosis by reducing the expression of TGF-*β*.	[120]
		Renal fibrosis	NaHS can inhibit the ERK- and *β*-catenin-dependent signaling pathways to improve renal fibrosis.	[121]
		Myocardial fibrosis	The chronic aerobic exercise or NaHS administration can downregulate myocardial hydroxyproline level and fibrotic area.	[125]
		Myocardial fibrosis	NaHS can reduce the content of Nox2/4, the phosphorylation of ERK1/2, and ROS, thereby reducing the myocardial fibrosis mediated by oxidative stress.	[115, 122]
		Myocardial fibrosis	NaHS inhibits the accumulation of extracellular matrix and increases blood vessel density to reduce myocardial fibrosis.	[124]
		Liver fibrosis	NaHS can elevate serum H_2_S level, decrease hyaluronic acid, and reduce the number of activated HSCs.	[131–133]
		Diabetic diaphragm fibrosis	NaHS can inhibit the inflammatory response mediated by NLRP3 inflammasome and reduce collagen deposition.	[137]
	GYY4137	Myocardial fibrosis	GYY4137 exerts antifibrosis and cardioprotective effects by enhancing the activation of endogenous natriuretic peptides after early ischemia.	[123, 126]
	SAC	Liver fibrosis	SAC can reduce liver fibrosis through its antioxidant and anti-inflammatory properties, as well as inhibiting the STAT3/SMAD3 signaling pathway.	[127]
	H_2_S	Idiopathic pulmonary fibrosis	H2S can inhibit the expression of NF-*κ*B p65 and downregulate Th2 cells to reduce fibrosis.	[135]

## Data Availability

The data used to support this study are included within the article.

## Abbreviations

3-MST: 3-Mercaptopyruvate sulfurtransferase
*α*-SMA: *α*-Smooth muscle actin
ATF2: Cyclic AMP-dependent transcription factor 2
ASK-1: Apoptosis signal-regulating kinase 1
AGE: Advanced glycation end-products
Atg7: Autophagy-related protein 7
CO: Carbon monoxide
CORMs: Carbon monoxide-releasing molecules
CCl_4_: Carbon tetrachloride
JNK: c-Jun N-terminal kinase
CO-HbV: CO-bound hemoglobin vesicles
CHF: Congenital hepatic fibrosis
CTGF/CCN2: Connective tissue growth factor
CINODs: Cyclooxygenase-inhibiting NO donors
CBS: Cystathionine-*β*-synthase
CSE: Cystathionine-*γ*-lyase
DAO: D-Amino acid oxidase
ET-1: Endothelin 1
EMT: Endothelial-mesenchymal transition
ECM: Extracellular matrix
FN: Fibronectin
GMCs: Glomerular mesangial cells
GSH: Glutathione
GS: Glycine-serine
HO: Heme oxygenase
HSCs: Hepatic stellate cells
HIV: Human immunodeficiency virus
H_2_S: Hydrogen sulfide
IPF: Idiopathic pulmonary fibrosis
Id1: Inhibitor of DNA binding 1
IGFBP: Insulin-like growth factor-binding proteins
KLF4: Krüppel-like factor 4
KLF2: Krüppel-like factor 2
LSECs: Liver sinusoidal endothelial cells
MMP: Matrix metalloproteinase
MEFs: Mouse embryonic fibroblasts
MI: Myocardial infarction
NOX: Nicotinamide adenine dinucleotide phosphate II (NADPH) oxidase
NLRP3: NOD-like receptor protein 3
NO: Nitric monoxide
NOS: NO synthase
NF-*κ*B: Nuclear factor-*κ*B
PD: Peyronie's disease
PAI-1: Plasminogen activator inhibitor-1
PDGF: Platelet-derived growth factor
ROS: Reactive oxygen species
SAC: S-Allyl-cysteine
SASP: Senescence-associated secretory phenotype
STAT6: Signal transducers and activators of transcription 6
SNAP: S-Nitroso-N-acetylpenicillamine
NaHS: Sodium hydrosulfide
Na_2_S: Sodium sulfide
SOD: Superoxide dismutase
TIMP1: Tissue inhibitor matrix metalloproteinase 1
TGF-*β*: Transforming growth factor *β*
TA: Tunica albuginea
UUO: Unilateral ureteral obstruction.
